# Perception of the Vegetation Elements of Urban Green Spaces with a Focus on Flower Beds

**DOI:** 10.3390/plants13172485

**Published:** 2024-09-05

**Authors:** Miroslav Poje, Anton Vukelić, Vesna Židovec, Tatjana Prebeg, Mihael Kušen

**Affiliations:** 1 Department of Ornamental Plants, Landscape Architecture and Garden Art, Faculty of Agriculture, University of Zagreb, Svetošimunska cesta 25, 10000 Zagreb, Croatia; vzidovec@agr.hr (V.Ž.); mkusen@agr.hr (M.K.); 2 Faculty of Humanities and Social Sciences, University of Zagreb, Ivana Lučića 3, 10000 Zagreb, Croatia

**Keywords:** ornamental plants, user preferences, vegetation volumes, vegetation plains, landscape design

## Abstract

Urban vegetation plays a crucial role in meeting the challenges posed by rapid urbanization and climate change. The presence of plants and green spaces in urban areas provides a variety of environmental, social, and economic benefits. Understanding how users perceive ornamental plants in public green spaces and what their preferences are for certain vegetation elements is extremely important for planning and designing functional and aesthetically interesting urban landscapes. Although landscape experts sometimes use their creativity to create new trends, it is important not to ignore the attitudes and preferences of the public, who sometimes have a different opinion from that of the experts. The aim of the study was to determine the perceptions and preferences of the public and landscape experts for different vegetation elements and the differences in attitudes between these two groups. The study was conducted in Croatia in April 2012 using an online survey (*n* = 348). The results showed that trees were the most preferred vegetation element and that the public preferred flower beds and lawns to a greater extent than the professionals. All respondents perceived vegetation elements as volumes (trees, shrubs, and hedges) and plains (flower beds and lawns). In addition, respondents perceived two basic types of flower beds according to the features that characterize them: conventional and sustainable. The results show that users perceive the functional and spatial characteristics of the different vegetation elements, which is very important for the design of functional and sustainable urban green spaces.

## 1. Introduction

Urban green spaces are important components of the urban environment and are critical to meeting the challenges posed by rapid urbanization and climate change. They provide significant environmental [1,2,3,4,5,6,7], psychological [8,9,10,11,12,13], social [14,15,16,17,18,19], and economic [20,21,22,23] benefits that improve the quality of life in urban areas. The strategic planning and management of urban green spaces is therefore crucial for strengthening the resilience and sustainability of cities.

The design and composition of urban green spaces, particularly the type and arrangement of vegetation elements such as trees, shrubs, lawns, hedges, and flower beds, have a significant impact on public perceptions and preferences. Understanding these perceptions and preferences is vital to designing urban green spaces that are more attractive, functional, and inclusive [24,25].

Perceptions of urban vegetation and preferences for certain types of vegetation are not uniform but vary between different demographic groups. Factors such as age, gender, cultural background, socioeconomic status, or personal experiences with nature play a decisive role in how urban greenery is perceived [26,27,28,29,30,31,32,33]. For example, older people prefer more accessible and well-maintained green spaces with sufficient seating and shade, while younger people prefer spaces that offer opportunities for physical activity and social interaction [34]. In some cultures, certain plants or green spaces have a symbolic meaning that increases their perceived value [35]. In addition, different cultures have different traditions and values regarding nature and landscape esthetics [36]. Personal experiences and familiarity with nature can also affect preferences for vegetation elements [37,38]. Perceptions and preferences are also influenced by expertise, i.e., the level of knowledge between the general public and experts [39,40,41,42].

People’s perceptions are influenced by various characteristics of vegetation, including species diversity, color, shape, and seasonal changes [43,44,45,46]. Trees are often the most conspicuous and appreciated vegetation elements in urban green spaces. People prefer the presence of trees in urban areas as they provide shade, reduce urban heat islands, and improve air quality [47,48]. Trees are also associated with aesthetic and psychological benefits, such as stress reduction and increased well-being [49]. The presence of mature, healthy trees can increase the attractiveness of green space and encourage more frequent use by the public [50]. Shrubs and understory vegetation contribute to the structural diversity and visual complexity of urban green spaces. These elements can form layers of vegetation that provide habitats for wildlife and increase biodiversity [51,52]. Shrubs are commonly used in landscaping to delineate spaces, create privacy, and provide seasonal interest through flowers and foliage. People generally perceive shrubs positively, especially when they are well maintained and strategically placed to enhance the aesthetics of urban spaces [53]. Hedges are often valued for their role in defining spaces, creating boundaries, and maintaining privacy. Hedges also serve functional purposes such as noise reduction, windbreaks, and providing habitat for birds and insects [54]. Properly designed green screens can also improve the aesthetic quality of streetscapes and public spaces. However, the perceptions of hedges can vary depending on their maintenance and species; poorly maintained hedges can be perceived as untidy and uninviting [55]. Flower beds are celebrated for their vibrant colors and seasonal interest and enhance the overall attractiveness of urban parks and gardens, making them one of the key elements in urban landscaping to improve aesthetics [56]. They serve as eye-catchers in gardens and parks, attracting attention and providing visual pleasure. The dynamic nature of flower beds with seasonally changing blooms can attract visitors and encourage repeat visits to urban green spaces [57]. Flower preferences are often influenced by the diversity and arrangement of plant species as well as the cultural and personal significance of the flowers used [58]. Flower beds are not only aesthetically pleasing but also contribute to urban biodiversity by providing habitats for pollinators [59]. Lawns serve as multifunctional areas for recreation, socializing, and relaxation in the urban environment [60]. People generally have a positive perception of lawns due to their versatility and the sense of openness they convey. Spending time in green play areas, which include lawns, can improve attention and reduce symptoms of attention deficit disorder in children [61]. However, the ecological value of lawns is limited compared to more diverse vegetation types [62]. The maintenance of lawns can be resource intensive and requires significant amounts of water, fertilizer, and pesticides [63]. Despite these drawbacks, cultural norms and expectations often lead to a preference for manicured lawns, reflecting ideals of order and tidiness in urban landscapes.

Despite the overwhelming benefits, urban vegetation is sometimes perceived negatively. Maintenance issues, safety concerns, and potential allergens are common challenges. Overgrown or poorly maintained green spaces can become havens for pests and criminal activity, leading to a sense of danger [64]. In addition, the presence of certain plants can aggravate allergies and cause discomfort and health problems in sensitive individuals [65]. Economic factors also play a role in negative perceptions. In low-income neighborhoods, green spaces can be underfunded and poorly maintained, exacerbating socioeconomic inequalities. These communities may perceive urban green space as a luxury rather than a necessity, especially when basic services are lacking [66,67].

It is important for urban planners and landscape architects to understand the perception of and preferences for vegetation elements in urban green spaces. By taking these preferences into account, they can design and manage green spaces. The integration of different vegetation elements can enhance the ecological value and aesthetic appeal of green spaces, increasing their use and appreciation by the public. Taking user preferences into account when designing green spaces can also encourage greater public engagement and stewardship. When people feel that their preferences and needs are taken into account, they are more willing to use and care for these spaces [68]. This participatory approach can lead to a more sustainable and resilient urban environment, as well-maintained and well-used green spaces contribute to the overall health and well-being of the community.

The objectives of this study were to identify preferences for the most common vegetation elements of public green spaces and their perceptions in a spatial context, identify preferences for the characteristics of flower beds and perceptions of different types of flower beds, and determine whether there are differences between preferences and perceptions between the general public and landscape experts.

## 2. Results

### 2.1. Perception and Preferences for Different Vegetation Elements of Green Spaces

Of the most common vegetation elements in public green spaces, respondents liked trees, flower beds, and lawns the most, while they liked shrubs and hedges to a relatively lesser extent (Table 1).

A *t*-test for two independent samples was used to test the statistical significance of differences in mean ratings of five selected vegetation elements of public green spaces between two types of respondents. No statistically significant difference was found in the mean results of trees, shrubs, and hedges between landscape experts and the general public.

A statistically significant difference was found between landscape experts and the general public in the evaluation of flower beds and lawns, with the public rating these two elements as more desirable than the experts (Table 2).

In an attempt to determine the latent dimensions, a factor analysis of the measurement factor was carried out according to the component model (Table 3).

The first factor, which explains 40.89% of the variance, is saturated by the following items: shrubs, hedges, and trees. Based on the content of the items, the first extracted factor can be interpreted as ‘vegetation volumes’.

The second factor is saturated by the following two items: flower beds and lawns. According to the content of the items, the second extracted factor is intended to measure the perception of ‘vegetation plains’ within green spaces. This factor explains 30.10% of the variance.

Two additively weighted indexes were constructed on the basis of the factor structure obtained, with the weights representing the factor loadings of the respective items on the reference factor. Although respondents perceived both types of structures positively, they showed a relatively greater preference for vegetation plains (Table 4).

A *t*-test was used to test the statistical significance of the differences between the average results of the public and the landscape experts for the weighted indexes. While no statistically significant difference was found between the average results of the public and the experts for the VVI (vegetation volumes index), a statistically significant difference was found in the average values for the VPI (vegetation plains index), with the general public showing a greater preference for flower beds and lawns than the experts (Table 5).

### 2.2. Importance of Certain Features of Flower Beds

The level of the arrangement was important for 87.3% of respondents, followed by the position of the flower bed in the space (71.8%) and the shape of the bed (71%). Of the features analyzed, the scent of the flowers was the least important to respondents. In fact, 21.8% of respondents said that the scent of the flowers was not important to them, and 31.3% of respondents said it was neither important nor unimportant.

The trends described were consistent with the average reference values of the characteristics analyzed. The average value of the most important feature of the flower bed according to the respondents (the level of the arrangement of the flower bed) was 4.29, while the average value of the least important feature (flower scent) was 3.38 (Table 6).

By factorizing the measurement factor, a statistically significant dimension was extracted to measure the importance of the selected features of the flower bed using the GK criterion. In other words, the factor is unidimensional as it measures only one item, which means that the evaluation of the importance of the individual flower bed features is not structurally differentiated (Table 7).

An additively weighted index was created based on the determined factor structure, whereby the scale weights represent the factor loadings of the respective items on the reference factor (Table 8).

A *t*-test was used to test whether there was a statistically significant difference between the mean values of the two groups of respondents with regard to the importance of the selected features of the flower beds.

No statistically significant difference was found between the mean values of the two groups in the evaluation of the importance of species diversity, the position of the flowerbed in the space, and the scent of the flowers.

A statistically significant difference was found in the evaluation of the importance of the color of the flowers, the shape of the flower bed, the height of the flowers, the change in the color of the bed over the course of the year, and the level of arrangement. For landscape experts, the color of the flowers, the shape of the bed, and the height and the change in the color of the bed over the course of the year are more important, while the general public places more importance on the level of the arrangement (Table 9).

### 2.3. Perception and Preferences for Certain Types of Flower Beds

Respondents liked multicolored flower beds with several types of flowers and those with a combination of low and tall flowers the most, while they liked monochrome flower beds and those with a single type of flower the least (Table 10).

In order to try to determine the latent dimensions, a factor analysis of the measurement factor was carried out. By factorizing the measurement factor, two statistically significant latent dimensions were extracted (Table 11).

The first factor is saturated by the following types of flower beds: with several types of flowers, multicolored, flower beds with a combination of low and tall flowers, and those characterized by organic shapes. According to the content of the items, the first extracted factor can be interpreted as a ‘sustainable flower bed’. This factor explains 32.25% of the variance.

The second factor, which explains 27.58% of the variance, is saturated by the following types of flower beds: monochromatic flower beds, those with one type of flower, and those with a geometric shape. Based on the content of the items, the first extracted factor can be interpreted as a ‘conventional flower bed’.

## 3. Discussion

### 3.1. The Perception of Vegetation Elements as Volumes and Plains

Of the vegetation elements most commonly found in green spaces, respondents preferred trees, flower beds, and lawns the most, while the group that liked shrubs and hedges was to a relatively lesser extent. As in numerous other studies, trees were the preferred vegetation element in public green spaces [69,70,71,72,73,74,75]. When comparing the results of the landscape experts and the general public, it was found that the latter preferred flower beds and lawns to a greater extent than the experts.

Factor analysis has identified two factors that influence the perception of vegetation elements: volumes and plains. Volumes include shrubs, hedges, and trees, while plains include flower beds and lawns. This division corresponds to the theoretical division of the basic elements of landscape design into point, line, volume, and plain [76,77,78]. Of course, this division can be considered in a broader context, as it includes all physical elements around us. In principle, any object or scene can be described with combinations of these elements. It is rare for an element to exist in isolation, and usually, they occur in various combinations [29,79].

The plain is defined as a fundamental, predominant, and inalienable component of the landscape [77]. The perception of vegetation as a plain emphasizes the visual and spatial aspects of green spaces. A well-manicured lawn in a park, for example, provides a large green space that conveys a sense of openness and continuity. The flatness of lawns provides a visually coherent background against which other elements of the landscape can stand out. Plains serve as platforms for recreational activities, gatherings, and leisurely strolls but sometimes also as focal elements (flower beds). Due to their two-dimensionality, their design possibilities are limited. Although they are not examined in this study, the category of vegetation plains could also include ground cover plants.

In contrast to the plains, the volumes are landscape elements that have a clearly pronounced or prominent third dimension, the height. Morphologically, they are relatively simple punctual phenomena or consist of densely grouped, often linear compositions such as avenues or hedges [77]. Trees, with their towering trunks and sweeping canopies, embody this concept and play a central role in defining the spatial experience of a landscape. Individual trees can form a focal point in the urban environment or in the landscape [80]. Their verticality adds vertical elements to the scenery, breaking the monotony of flat surfaces and creating diverse micro-environments beneath their canopies. Shrubs and hedges may be smaller, but they too have a certain volume that occupies a three-dimensional space.

The general public showed a greater preference for vegetation plains, i.e., flower beds and lawns. There are several hypothetical explanations for this result. First, it could be that the public perceives the above elements as ones that are appropriately maintained. After all, most flower beds with seasonal flowers have a representative character and are therefore regularly maintained, regardless of whether they are located in the city center or in more remote parts of the city. The lawns are mowed regularly and are therefore also characterized by a high degree of cleanliness. This suggests that the public prefers a more conventional design with an emphasis on a neat and tidy appearance, which is also suggested by other studies [44,81]. On the other hand, it is assumed that the experts consider flower beds and lawns to be too simple elements of public green spaces. Furthermore, the experts are more critical in their assessments because the overall context is important to them so they do not tend to single out individual elements from the whole.

By looking at vegetation in terms of plains and volumes, landscape planners and architects can better understand and influence the spatial qualities of green spaces. They can use this perspective to create balanced and harmonious compositions that effectively integrate both flat surfaces and vertical elements. By strategically placing flat plains of vegetation next to voluminous trees and shrubs, designers can manipulate spatial relationships, control views, and evoke specific emotional responses. This encourages consideration of scale, texture, and rhythm in the landscape, creating a varied and visually appealing environment. This view also contributes to sustainable practices by encouraging the conservation of existing vegetation and the careful incorporation of new planting. By recognizing the dual nature of vegetation—its expansive flatness as plains and its dynamic three-dimensionality as volumes—we gain insight into the spatial, visual, and functional aspects of landscapes. This perception not only influences landscape design and planning, but also fosters a deeper connection to nature, enriches our experience of green spaces, and promotes environmental stewardship.

### 3.2. The Perception of Certain Features of Flower Beds

The degree of arrangement proved to be the most important feature of the flower beds studied, while the scent of the flowers was the least important to the respondents. While the color of the flowers, the shape of the bed, the height, and the change in color of the bed over the course of the year are more important to the expert, the level of arrangement is more important to the general public. This can possibly be explained by the perceptual orientation of the respondents, with the general public paying the most attention to the visual-aesthetic impression and maintenance requirements when evaluating a flower bed, while the expert public attaches more importance to other form and functional aspects of a flower bed.

In the context of preferences for the color and height of flowers, Todorova et al. [57] indicate that the height of flowers is more important to respondents than the color, while this study came to different conclusions. In this study, a statistically significant difference was found between the assessment of the importance of flower color and flower height (t _(347)_ = 2.198, *p* = 0.029). The difference in the results can be explained in two ways. First, Todorova and colleagues’ study was based on visual preferences, i.e., photomontage simulations were used for the study, whereas the present study was based on rating preferences for abstract conceptual categories. Moreover, in the aforementioned study, respondents were shown *Althea rosea* L., a flower species of exceptional height (1.5 to 2.5 m), but which can be evaluated as a species with weaker aesthetic qualities, even if this is a subjective impression. In other words, there is a possibility that different results would have been obtained if respondents had been offered a different flower of the same height but with more aesthetic qualities.

### 3.3. The Perception of Flower Beds as Conventional and Sustainable

The survey revealed that respondents liked flower beds with multiple flower varieties, multi-colored beds, and those with a combination of low and tall flowers the most, while they liked monochromatic flower beds with a single flower variety the least. Based on the expressed preferences regarding the features that characterize the types of flower beds, the results showed that respondents perceive two types of flower beds that can be interpreted as sustainable and conventional. This categorization was also mentioned in another study that examined the sustainability of flower beds with perennials [82].

Sustainable flower beds are characterized by a variety of flower types, which usually means more colors and different heights of flowers as well as an organic shape. Conventional flower beds, on the other hand, are characterized by the monochrome use of a single type of flower and a geometric shape. Although the shape of a flower bed itself is not a measure of its conventionality or sustainability, it is an indication of respondents’ perceptions, with a preference for an organic shape indicating a preference for more natural flower beds that are inherently more sustainable. On the other hand, regularity of shape does not necessarily define a flower bed as conventional, as a sustainable flower bed can also have a geometric shape. Nevertheless, the results suggest that respondents associate such a shape with beds of seasonal flowers, which are most commonly found in green spaces and have a conventional character.

Currently, the guidelines for the design of flower beds in urban areas are based on the assumption that citizens like them as they are seasonal flowers. Since such flower beds are conventional in nature, the results of this research suggest that the public is more inclined towards a sustainable type of flower beds because users prefer diversity and species richness [83,84,85,86]. With this in mind, it is necessary to reconsider the appropriateness of the practice that still favors flower beds with seasonal flower species rather than using more perennials that would make them aesthetically acceptable but immeasurably more sustainable.

### 3.4. Limitations of the Study

The main limitation of this study is a convenience sample. Another limitation is the gender structure of the respondents. Although the higher participation of women in the survey can be explained by the fact that they are more inclined to complete surveys [87] and that most experts were female at the time of the survey, the results were verified in a later procedure. The Rim Iterative Method (RIM) was used for weighting in order to compare the socio-demographic structure of the realized sample (gender structure of respondents) with the parameters of the population in order to ensure the representativeness of the sample for the reference target population. Data from the national statistical office were used for the weighting. Therefore, the trends observed in the realized sample can serve as an indicator of the population parameters.

## 4. Materials and Methods

### 4.1. Sample

The study was conducted in Croatia in April 2012 on a convenient sample (*n* = 348) using the online survey method. The advantages and disadvantages of the online survey method have been discussed by many authors [88,89,90].

The sample comprised a proportion of the general (non-professional) public (*n* = 243) and a proportion of landscape experts (*n* = 105). This sample was selected to determine the difference between the perceptions and preferences of experts and the general public.

### 4.2. Data Collection

The invitation to participate in the study was sent to landscape experts via the Croatian Society of Landscape Architects and the Croatian Chamber of Architects-Committee for Landscape Architecture, while the invitation to the general public was sent via e-mail and social networks. The data collection period for this survey was April 2012. The survey took about 15 min to complete. The answers were collected using the SurveyGizmo platform. The responses were automatically saved in a .csv file and then imported into the SPSS software for processing. All participants gave their informed consent to take part in the study and participation was completely voluntary.

### 4.3. Socio-Demographic Characteristics of the Respondents

The sample comprised 348 people, of whom 68 were male (19.5%) and 280 were female (80.5%). In terms of age structure, the respondents were divided into three groups: up to 25 years old (*n* = 151), from 26 to 35 years old (*n* = 138), and over 35 years old (*n* = 59). In terms of the education level, the sample did not include any respondents with a lower level of education. Thus, 42.2% of respondents had completed secondary education, 6.9% of respondents had completed upper secondary education, 41.7% of respondents had completed tertiary education, and 9.2% of respondents had completed postgraduate studies (Masters or PhD). Of the total number of respondents, 50.3% were employed, 4.9% were unemployed, 43.4% were students, and 1.4% were retired. The self-assessed wealth status of the immediate family was rated as very high by 4.9% of respondents, high by 28% of respondents, average by 57.5% of respondents, low by 9.2%, and very low by 0.6%.

### 4.4. Survey Questionnaire

The questionnaire consisted of the following factors:

- A factor measuring preferences for individual vegetation elements of public green spaces.
- A factor measuring the importance of certain features of flower beds.
- A factor measuring preferences for certain types of flower beds.
- A series of questions measuring the socio-demographic characteristics of respondents.

The factor for measuring preferences for individual vegetation elements in public green spaces consisted of five groups that were offered for evaluation: trees, shrubs, hedges, flower beds, and lawns. A five-point rating scale was assigned to the evaluation groups, with the lowest value (1) indicating that the subjects did not like the content offered for evaluation at all, while the highest value on the scale (5) indicated that the subjects liked the content offered for evaluation very much.

The factor for measuring the importance of certain characteristics of flower beds consisted of eight groups: the color of flowers, variety of flower species, the shape of the flower bed, position of flower bed in space, height of flower species, scent of flowers, level of arrangement, and change in color of flowerbed over the year. The groups were indicated on an ordinal rating scale with five levels. The lowest value (1) meant that the feature of the flower bed was not at all important to the respondents, while the highest value (5) meant that the feature was very important to the respondents.

The factor for measuring preferences for certain types of flower beds consisted of ten groups expressing the shape of the flower bed (a geometric shape, an organic shape, and a combination of geometric and organic shapes), the variety of flower species in the bed (flower beds with one flower species, flower beds with several flower species), the height of the flower species in the flower bed (a combination of tall and low flowers), and the color of the flowers in the bed (monochromatic flower beds, colorful flower beds). The groups were assigned an ordinal rating scale with five levels. The lowest value (1) meant that the respondents did not like the group offered at all, while the highest value (5) meant that the respondents liked the group offered very much.

In addition, certain socio-demographic characteristics of the respondents were examined. A series of questions to measure the aforementioned characteristics included the following variables: gender, age, education level, work status, and wealth status of the respondent’s immediate family.

The measurement factors (factor for measuring preferences for certain vegetation elements of public green spaces, factor for measuring the importance of certain features of flower beds, and factor for measuring preferences for certain types of flower beds) were subjected to the factor analysis procedure. The initial solutions were subjected to orthogonal transformation using the Varimax rotation criterion in the context of multidimensional measures, while the final factor structure was guided by the attempt to comply with the principle of Thurstone’s simple structure. Based on the factor solutions obtained, an appropriate number of additive weighted indexes were constructed for each measurement factor. In the case of a unidimensional measurement item, one index was constructed, while in the case of a multidimensional measurement item, as many indexes were constructed as statistically significant latent dimensions were extracted. At the same time, the weights of the indicators were represented by the factor loadings of the individual indicators on the latent reference dimension.

### 4.5. Statistical Analysis

The data were analyzed using IBM SPSS Statistics software (version 27). The data were analyzed to identify differences between the landscape experts and members of the general, non-expert public.

The empirical data were analyzed using the methods and procedures of descriptive, inferential, and multivariate statistics. Within the framework of descriptive statistics, the variables were analyzed using univariate techniques and appropriate descriptive statistical indicators (frequency distributions, percentage distributions of responses, mean modal and median values, and standard deviation). As part of the inferential statistical analysis of the data, a *t*-test was performed for two independent samples [91]. The multivariate technique (principal component analysis) was used to test the factor structure of a multilevel factor measuring preferences for different types of vegetation elements, the importance of different features of flowerbeds, and perceptions and preferences for different types of flower beds. The factor solution was determined using the GK criterion to extract statistically significant latent dimensions [92] and was subjected to an orthogonal transformation using the Varimax rotation criterion [93].

All statistical tests were performed at a 5% level of significance.

## 5. Conclusions

Users’ preferences for urban vegetation are shaped by a complex interplay of environmental, psychological, and social factors. Trees, shrubs, hedges, flower beds, and lawns each offer unique benefits and pose different challenges in urban landscapes. By understanding and accommodating these preferences, urban planners and designers can create green spaces that improve the livability and sustainability of cities and ultimately contribute to the well-being of city dwellers. The integration of different vegetation types combined with community engagement is key to successful urban greening initiatives that match the preferences and values of the population.

## Figures and Tables

**Table 1:** Selected descriptive statistical indicators of the analyzed vegetation elements of public green spaces (*n* = 348).

Vegetation Elements	Mean	Mdn	Mod	SD
Trees	4.48	5	5	0.68
Flower beds	4.16	4	5	0.91
Lawns	4.15	4	5	0.94
Shrubs	3.79	4	4	1.02
Hedges	3.56	4	4	1.03

**Table 2:** Preferences for vegetation elements where a statistically significant difference was found between the average results of landscape experts and the general public (*n* = 348).

Variable	Public Type	*n*	M	SD	F	t
Flower beds	General public	243	4.35	0.77	11.241 **	t_(157)_ = 5.806 ***
	Landscape experts	105	3.70	1.03		
	Total	348	4.16	0.91		
Lawns	General public	243	4.42	0.80	4.344 *	t_(168)_ = 8.424 ***
	Landscape experts	105	3.52	0.96		
	Total	348	4.15	0.94		

Note: *, significant at *p* < 0.05; **, significant at *p* < 0.01; ***, significant at *p* < 0.001.

**Table 3:** Factor analysis of the different vegetation elements of green spaces.

Items	Factor Loadings	
	1	2
Shrubs	0.849	
Hedges	0.808	
Trees	0.770	
Flower beds		0.871
Lawns	0.263	0.812
Eigenvalue	2.04	1.51
% of variance	40.89	30.10
Total explained variance	86.17%	

**Table 4:** Selected descriptive statistical indicators of the weighted indexes.

Index	Min	Max	M	Mdn	Mod	SD	Skewness	Kurtosis	M*
Vegetation volumes	3.20	12.14	9.54	9.71	10.48	1.85	−0.69	0.15	3.94
Vegetation plains	1.68	8.42	6.99	7.54	8.42	1.34	−1.09	1.01	4.16

Note: M*, The average value of the arithmetic means of the reference particles.

**Table 5:** The difference in the average preference for vegetation plains between two types of respondents (*n* = 348).

Public Type	*n*	M	SD	F	t
General public	243	4.39	0.66	11.009 **	t _(165)_ = 8.565 ***
Landscape experts	105	3.61	0.81		

Note: **, significant at *p* < 0.01; ***, significant at *p* < 0.001.

**Table 6:** Selected descriptive statistical indicators of the analyzed characteristics of flower beds (*n* = 348).

Characteristics of the Flower Beds	M	Mdn	Mod	SD
The level of the arrangement	4.29	4	5	0.86
The shape of the bed	3.86	4	4	1.08
The position of the bed in the space	3.82	4	4	1.02
The color of the flowers	3.73	4	4	1.11
Changing colors of the flower bed throughout the year	3.60	4	5	1.27
Variety of flower species	3.58	4	4	1.07
Height of the flowers	3.54	4	4	1.13
The scent of the flowers	3.38	3	3	1.16

**Table 7:** Factor analysis of the importance of selected features of flower beds.

Items	Factor Loadings
	1
The shape of the bed	0.768
The position of the bed in the space	0.743
Height of the flowers	0.659
Color of the flowers	0.650
Changing colors of the flower bed throughout the year	0.635
Variety of flower species	0.606
The level of the arrangement	0.469
The scent of the flowers	0.443
Eigenvalue	3.18
Total explained variance	39.81%

**Table 8:** Selected descriptive statistical indicators of the weighted index (*n* = 348).

Index	Min	Max	M	Mdn	Mod	SD	Skewness	Kurtosis	M*
The importance of the characteristics of flower beds	4.97	24.87	18.53	18.77	24.87	3.49	−1.28	3.07	3.73

Note: M*, The average value of the arithmetic means of the reference particles.

**Table 9:** Indicators for the conducted test of the difference in the average importance of selected features of the flower bed between two groups of respondents (*n* = 348).

A Characteristics of Flower Beds	Public Type	*n*	M	SD	F	t
Color of the flowers	General public	243	3.63	1.14	7.488 **	t_(220)_ = 2.504 *
	Landscape experts	105	3.94	1.02		
	Total	348	3.73	1.11		
The shape of the bed	General public	243	3.66	1.13	12.511 ***	t_(271)_ = 6.209 ***
	Landscape experts	105	4.32	0.80		
	Total	348	3.86	1.08		
Height of the flowers	General public	243	3.35	1.15		
	Landscape experts	105	3.98	0.96	10.379 **	t_(233)_ = 5.264 ***
	Total	348	3.54	1.13		
The level of the arrangement	General public	243	4.46	0.80		
	Landscape experts	105	3.89	0.85	0.130	t_(346)_ = 6.028 ***
	Total	348	4.29	0.86		
Changing colors of the flower bed throughout the year	General public	243	3.39	1.31		
	Landscape experts	105	4.10	1.01	20.111 ***	t_(252)_ = 5.416 ***
	Total	348	3.60	1.27		

Note: *, significant at *p* < 0.05; **, significant at *p* < 0.01; ***, significant at *p* < 0.001.

**Table 10:** Selected descriptive statistical indicators of preference for certain types of flower beds (*n* = 348).

Characteristics of the Flower Bed Types	M	Mdn	Mod	SD
With more types of flowers	4.00	4	4	0.75
Multicolored flower bed	3.99	4	4	0.80
A combination of low and tall flowers	3.81	4	4	0.97
Organic shape	3.53	4	4	0.88
Geometric shape	3.48	4	4	0.89
With a single type of flower	3.47	4	4	0.90
Monochrome flower bed	3.36	4	4	0.91

**Table 11:** Factor analysis of the typology of flower beds according to respondents’ preferences (*n* = 348).

Items	Factor Loadings	
	1	2
With more types of flowers	0.872	
Multicolored flower bed	0.861	
A combination of low and tall flowers	0.670	
Organic shape	0.543	
Monochrome flower bed		0.894
With a single type of flower		0.827
Geometric shape		0.621
Eigenvalue	2.26	1.93
% of variance	32.25	27.58
Total explained variance	59.84%	

## Data Availability

The data presented in this study are available upon request from the corresponding author.
